# Qualitative and quantitative evaluation of Fetal Bovine Serum composition: toward ethical and best quality *in vitro* science

**DOI:** 10.1016/j.namjnl.2025.100047

**Published:** 2025-09-07

**Authors:** Ana Clara Silva Stival, Artur Christian Garcia da Silva, Marize Campos Valadares

**Affiliations:** Laboratory of Education and Research *In vitro* Toxicology – Tox In, Faculty of Pharmacy, Federal University of Goiás, Goiânia, GO, Brazil

**Keywords:** Alternative animal models, Fetal bovine serum, *In vitro* sciences, Cell culture

## Abstract

Fetal bovine serum (FBS) is widely used as a supplement in cell culture due to its complex composition, which supports cell growth, and metabolism for conducting experiments with mammalian cells *in vitro*. However, concerns regarding reproducibility, ethical considerations, and batch variability have prompted efforts to investigate its biochemical composition and potential alternatives, to subsidize the development of chemically defined alternatives. This study aimed to analyze the qualitative and quantitative composition of different FBS samples, including growth factors, vitamins, hormones, lipids, proteins, ions, and organic compounds. Additionally, mycoplasma contamination was assessed. FBS samples from different suppliers (Brazil, USA, and Paraguay) were evaluated before and after inactivation at 56 °C for 30 min. Results demonstrated the absence of mycoplasma in all samples. Among the 58 biochemical parameters analyzed, 20 exhibited significant variability (16–102 %) in non-inactivated samples, while 19 parameters showed variations (16–84 %) after inactivation. The highest variability was observed for luteinizing hormone and transferrin. Growth factor analysis revealed that epidermal growth factor, and insulin-like growth factor type 1 concentrations were below detection limits, whereas basic Fibroblast Growth Factor, and vascular endothelial growth factor A concentrations considerably reduced after the heat inactivation process for most of the evaluated samples. These findings highlight the heterogeneity of FBS composition, which may impact reproducibility in cell-based assays. Despite being considered a universal cell culture supplement, our results demonstrate that alternatives to FBS should be explored to improve the *in vitro* data quality and move towards a more ethical science in the field of new approach methodologies.

## 1 Introduction

In the field of new approach methodologies (NAM) cell and tissue culture are the main tools that allow the generation of data aiming to replace the use of living animals. However, to keep cells viable and metabolically active *in vitro*, fetal bovine serum (FBS) is widely employed as a default additive to culture media. FBS is a complex mixture used in nearly all cell cultures as a supplement to maintain cell viability and proliferation, both during routine culture and cryopreservation, due to its capacity to support cellular metabolism over extended periods (Van der Valk et al. 2010). Serum is still the most widely used animal-derived product in cell cultures, and its composition includes growth factors, hormones, amino acids, proteins, carbohydrates, lipids, vitamins, non-protein nitrogen, inorganic salts, and antibodies (Cassotta et al. 2022).

On the other hand, the use of FBS also has numerous disadvantages for cell culture, especially when observing the dynamics of modern science and the urgency for reproducibility in science (Hartung, 2017). Moreover, ethical concerns are associated with its use, as FBS is derived from the blood of fetal calves collected from pregnant cows during slaughter at abattoirs. Refined science also seeks animal welfare that largely demands changes in methodologies toward animal-free models (Lee et al. 2022).

The quality parameters in the reproducibility of *in vitro* assays and the heterogeneity in terms of composition of the FBS can influence the cellular responses, and consequently the reproducibility of *in vitro* results. In 2016, it was estimated that only 70 % of the research generated in the world would have reproducible results and methodologies (Baker, 2016). Specifically in the context of *in vitro* assays, several challenges have been reported, particularly regarding cell culture-based methods, which often suffer from poor interlaboratory reproducibility. This lack of consistency significantly impacts pharmacological, toxicological, and broader biomedical research applications (Hirsch and Schildknecht, 2019).

The absence of qualitative and quantitative standards for animal-derived products, particularly FBS, can significantly affect experimental outcomes. This variability may stem from multiple factors, including the animals’ geographic origin, diet, and the environmental and climatic conditions to which they are chronically exposed (Baker, 2016; Price and Gregory, 1982; Van der Valk et al. 2010).

Although detailed research on the composition of FBS remains limited, some studies have demonstrated that it differs from the blood or serum of adult animals. This compositional difference is likely one of the main reasons why FBS has not yet been successfully replaced and continues to contribute to variability in biotechnological applications (Lee et al. 2022).

FBS functionality is also attributed to the presence of specific components, which includes growth factors such as epidermal growth factor (EGF), insulin-like growth factor type 1 (IGF-1), basic Fibroblast Growth Factor (bFGF), vascular endothelial growth factor A (VEGF-A) (Zheng et al., 2006), as well as physicochemical properties like viscosity and pH (Brunner, 2010). However, concerns remain regarding its batch-to-batch variability, ethical sourcing issues, and the presence of undesirable components, including bilirubin, hemoglobin, complement proteins, trace elements, and antibodies (Coecke et al., 2005). Heat inactivation is commonly applied to reduce immunogenicity by denaturing complement proteins (Nims and Harbell, 2017), but it may also degrade heat-sensitive components such as growth factors and enzymes, potentially compromising serum functionality. Therefore, evaluating the biochemical effects of this process is crucial for improving experimental reproducibility.

Replacement of FBS has been proposed, but still most of serum replacements are based on animal derivatives, such as, serum from adult bovine, pig, horse, goat, human platelet lysate (hPL), chicken embryo pituitary extracts, and bovine milk fractions (Belford et al. 1995; Klagsbrun, 1980; Pakkanen and Neutra, 1994; Steimer et al. 1981). All of them have the same problems of undefined compositions and low uniformity of the batches.

Thus, a rational approach to developing effective FBS replacements involves addressing the specific needs of different cell lines. Nevertheless, the precise composition of FBS is not fully disclosed in the literature, particularly with respect to the concentrations of hormones and growth factors. These components are critical for the biological activity of FBS and for designing adequate replacement strategies.

Based on these considerations, the aim of this study was to perform a comprehensive qualitative and quantitative evaluation of different commercial FBS products, including their content of growth factors, vitamins, hormones, lipids, proteins, ions, and organic compounds. By highlighting the significant variability among FBS suppliers and the impact of heat inactivation on their composition, we intend to provide additional evidence of the inconsistencies inherent to this supplement. These findings are useful as a foundational dataset to support the development of chemically defined, serum-free media, contributing to increased reproducibility and ethical standards in cell-based research.

## 2 Experimental procedures

### 2.1 Sample preparation

Six commercial FBS samples from three different geographic origins (Brazil, Paraguay, and USA) were analyzed. For each country, products from distinct suppliers were selected. Specifically, Samples 1, 2, and 3 correspond to FBS (Brazil) from suppliers A, B, and C; Samples 4 and 5 to FBS (USA) from suppliers A and B; and Sample 6 to FBS (Paraguay) from supplier A. To preserve confidentiality, brand names and catalog numbers are not disclosed. All samples were sourced from certified suppliers operating under Good Manufacturing Practices. Notably, suppliers A and B provided products in more than one country, allowing us to examine potential geographic variation in addition to inter-supplier variability.

Samples were coded and aliquoted, with one portion subjected to heat inactivation using the standard protocol: incubation in a water bath at 56 °C for 30 min. This procedure is intended to inactivate complement proteins and minimize immune-like responses in cell culture. However, it may also alter concentrations of heat-sensitive biomolecules such as hormones, enzymes, and growth factors. Therefore, both heat-inactivated and non-inactivated samples were analyzed to assess the impact of this treatment on their composition.

All samples were also evaluated for mycoplasma contamination. For thawing, only specific volumes were processed using a controlled water bath at 37 °C to ensure single freeze-thaw cycles, thereby preserving sample integrity. Controlled thawing is essential to maintain physicochemical stability, as rapid or uncontrolled thawing can lead to protein denaturation, precipitation, or aggregation, particularly of labile components such as growth factors and hormones.

After processing, samples were stored at 2–8 °C for a maximum of 24 h prior to transport for analysis and testing.

### 2.2 Mycoplasma analysis

The mycoplasma analysis was performed using the MycoAlert™ PLUS Mycoplasma Detection Kit (Lonza, Walkersville, USA, LT07–701) according to the manufacturer’s instructions. Both inactivated and non-inactivated samples were tested following the specified inactivation procedure. For quality control, the MycoAlert™ Assay Control Set (Lonza, Walkersville, USA, LT07–518) was used as positive and negative controls. All measurements were recorded using a Fluostar Omega fluorimeter (BMG Labtech).

### 2.3 FBS analysis of vitamins, hormones, lipids, proteins, ions, carbohydrates, and organic compounds

The selection of biochemical parameters was guided by the availability of validated assays routinely employed in clinical laboratories. These tests provided a broad overview of relevant classes of biomolecules, including proteins, enzymes, hormones, and antibodies, enabling comprehensive comparative analysis of FBS composition. The inclusion of anti-thyroglobulin antibody was based on its availability in standard panels rather than specific immunological interest. At this stage, we screened three different products aiming to check variations in the composition regarding different countries (Brazil and USA) and suppliers (A and B).

For the biochemical analysis, samples were transported in sterile collection tubes immediately before the assays. A total of 58 parameters relevant for the identification and quantification of FBS constituents were analyzed. The analysis of inactivated and non-inactivated FBS samples included the identification and quantification of:

• Vitamins: Vitamin A, vitamin B12, vitamin C, vitamin D3, and vitamin E.
• Hormones: 17-alpha hydroxyprogesterone, insulin, total T3, thyroxine (T4), free T4, progesterone, estradiol, basal cortisol, growth hormone (GH), luteinizing hormone (LH), prolactin, follicle-stimulating hormone (FSH), and parathormone (PTH).
• Lipids: high-density lipoprotein (HDL), total cholesterol, and fractions, low-density lipoprotein (LDL), and triglycerides.
• Proteins: Albumin, globulin, folic acid, amylase, cholinesterase, serum creatinine, alkaline phosphatase, gamma-glutamyl transferase (GGT), lactate dehydrogenase (LDH), lipase, thyroxine (T4), free T4, pyruvate glutamate transaminase (TGP), glutamic oxaloacetic transaminase (TGO/AST), transferrin, and total proteins.
• Ions: Calcium, chlorine, iron, magnesium, potassium, and sodium.
• Carbohydrates: Glucose and fructosamine.
• Organic Compounds: Lactic acid, adrenocorticotropic hormone (ACTH), ammonia, direct bilirubin, indirect bilirubin, total bilirubin, sodium, urea, and uric acid.

All the analyses were performed using commercial kits according to the manufacturer's instructions. The 58 measured parameters, along with the methods, equipment, and measurement units are detailed in Table 1.

**Table 1:** Description of the methods used for the characterization of FBS components, including the respective brands, equipment, and measurement units for each analyzed constituent.

Tests	Method and Brand	Equipment	Units
17- alpha hydroxyprogesterone	Chemiluminescence	–	**ng/dL**
Folic acid	Chemiluminescence, Abbott diagnostics	Architect i1000	**ng/mL**
Lactic acid	Colorimetric	–	**mmol/L**
Uric acid	Enzymatic, Trinder Labtest	LabMax 240	**mg/dL**
Adrenocorticotropic hormone	Electrochemiluminescence	–	**pg/mL**
Albumin	Biuret and bromocre greenol, Labtest	LabMax 240	***g*/dL**
Amylase	Substratecnpg3, Labtest	LabMax 240	**U/L**
Ammonia	Enzymatic	–	**umol/L**
Anti-thyroglobulin antibody	Chemiluminescence, Abbott diagnostics	Architect i1000	**UI/mL**
Bilirubin	Labtest dca, Labtest	LabMax 240	**mg/dL**
Direct bilirubin	Labtest dca, Labtest	LabMax 240	**mg/dL**
Indirect bilirubin	Labtest dca, Labtest	LabMax 240	**mg/dL**
Calcium	Arsenazo iii, Labtest	LabMax 240	**mg/dL**
Iron fixing capacity latent	Labtest ferrozine, Labtest	LabMax 240	**mcg/dL**
Iron fixing capacity saturation	Labtest ferrozine, Labtest	LabMax 240	**mcg/dL**
Iron fixing capacity - total	Labtest ferrozine, Labtest	LabMax 240	**mcg/dL**
Chlorine	Mercury thiocyanate, Labtest	LabMax 240	**meq/L**
Total cholesterol and fractions	Colorimetric and enzymatic, Labtest	LabMax 240	**mg/dL**
Cholinesterase	Colorimetric	–	**mg/dL**
Basal cortisol	Chemiluminescence, Abbott diagnostics	Architect i1000	**micro/dL**
Serum creatinine	Labtest	LabMax 240	**mg/dL**
Estradiol	Chemiluminescence, Abbott diagnostics	Architect i1000	**pg/mL**
Iron	Labtest ferrozine, Labtest	LabMax 240	**Ug/dL**
Alkaline phosphatase	Bowers e mc comb modifiedo, Labtest	LabMax 240	**U/L**
Phosphor	Daly e ertingshausen, Labtest	LabMax 240	**mg/mL**
Fructosamine	Colorimetric	–	**mUI/mL**
Follicle-stimulating hormone	Chemiluminescence, Abbott diagnostics	Architect i1000	**mIU/mL**
Gamma-glutamyl transpeptidase	Szasz modified, Labtest	LabMax 240	**U/L**
Growth hormone	Chemiluminescence liaison	–	**ng/mL**
Glucose	God, trinder, Labtest	LabMax 240	**uUmL**
Globulin	Biuret and bromocre green, Labtest	LabMax 240	***g*/dL**
High density lipoprotein	Colorimétrico e enzimático, Labtest	LabMax 240	**mg/dL**
Insulin	Chemiluminescence, Abbott diagnostics	Architect i1000	**uU/mL**
Lactate dehydrogenase	Colorimetric and enzymatic, Labtest	LabMax 240	**U/L**
Low density lipoprotein	Colorimetric and enzymatic, Labtest	LabMax 240	**mg/dL**
Luteinizing hormone	Chemiluminescence, Abbott diagnostics	Architect i1000	**mUI/mL**
Lipase	Automated enzymatic colorimetric, Cobas 501	–	**UI/L**
Magnesium	Labtest	LabMax 240	**mg/dL**
Potassium	Selective ion electrolyte analyzer, we-301, Wama diagnósticos	–	**mmol/L**
Progesterone	Chemiluminescence, Abbott diagnostics	Architect i1000	**ng/mL**
Prolactin	Chemiluminescence, Abbott diagnostics	Architect i1000	**ng/mL**
Total proteins	Biuret and bromocre green, Labtest	LabMax 240	***g*/dL**
Parathyroid hormone	Chemiluminescence, Abbott diagnostics	Architect i1000	**pg/mL**
Sodium	Selective ion electrolyte analyzer, we-300	Wama Diagnósticos	**mmol/L**
Total triiodothyronine	Chemiluminescence, Abbott diagnostics	Architect i1000	**ng/mL**
Thyroxine	Chemiluminescence, Abbott diagnostics	Architect i1000	**mcg/dL**
Glutamic oxaloacetic transaminase	Kinetics uv-ifcc, Labtest	LabMax 240	**U/L**
Glutamic pyruvic transaminase	Kinetics uv-ifcc, Labtest	LabMax 240	**UI/L**
Transferrin	Immunoturbidimetry, Labtest	LabMax 240	**mg/dL**
Triglycerides	Colorimetric and enzymatic, Labtest	LabMax 240	**mg/dL**
Urea	UV enzymatic, Labtest	LabMax 240	**mg/dL**
Vitamin A	UPLC - in house method	–	**mg/L**
Vitamin B12	Chemiluminescence, Abbott diagnostics	Architect i1000	**pg/mL**
Vitamin C	HPLC ms/ms	–	**mg/L**
Vitamin D3	Chemiluminescence, Abbott diagnostics	Architect i1000	**ng/mL**
Vitamin E	HPLC	–	**mg/L**

### 2.4 Measurement of growth factors in FBS samples

The quantification of growth factors in both inactivated and non-inactivated FBS samples was evaluated. To enhance detection, even at low concentrations, the samples were subjected to lyophilization. This process was carried out using a programmed cycle that included three main stages: freezing, primary drying, and secondary drying. Lyophilization was incorporated into the sample preparation to concentrate growth factors and ensure accurate quantification. In contrast to the broader biochemical analyses (which included three FBS samples), the growth factor evaluation was extended to six commercial FBS samples from different suppliers and country origin available in our laboratory at the time of analysis. This decision aimed to increase the representativeness and robustness of the comparison regarding bFGF and VEGF-A concentrations. All samples were subjected to the same lyophilization and ELISA protocols to ensure consistency.

A 10 mL aliquot of each sample remained in the lyophilizer (Thermo Electron Corporation, MicroModulyo, Freeze Dryer) for 48 h to ensure complete water removal. Immediately before analysis, the lyophilized material was reconstituted in 1.5 mL of sterile PBS (phosphate buffered saline solution without calcium and magnesium). No spike-in controls containing known quantities of growth factors were included to evaluate potential losses due to lyophilization. The lyophilization and reconstitution steps were applied identically across all FBS samples, ensuring internal consistency for comparative purposes. Therefore, the reported concentrations should be interpreted as relative values, suitable for assessing inter-product variability rather than determining absolute protein levels.

The specific growth factors were analyzed using ELISA immunoenzymatic detection specific assays, including EGF (RAB0149, lot 1202I0115), IGF-1 (RAB1187, lot 0921I0659), bFGF (RAB1184, lot 1012I066), and VEGF-A (RAB1197, lot 1012I0660), all obtained from Millipore Sigma (Saint Louis, MO, USA). Quantification was performed according to the manufacturer’s protocols. ELISA readings were conducted at a wavelength of 450 nm using a plate-reading spectrophotometer (Multiskan Spectrum, Thermo Scientific, Waltham, MA, USA).

### 2.5 Statistical analysis

The results were presented as the mean ± standard deviation. All biochemical analyses were performed in technical triplicates (*n* = 3 per sample), using a single batch per FBS supplier. Each replicate corresponds to an independent measurement of the same batch. For the biochemical tests, a one-way ANOVA followed by Tukey’s post hoc test was used to compare the FBS samples composition before and after heat inactivation. The comparisons between inactivated and non-inactivated samples were performed using the Student’s *t-*test. Statistical analyses were performed using GraphPad Prism 10.0 software.

## 3 Results

### 3.1 Presence of mycoplasma in the FBS samples

The identification and quantification of mycoplasma in both inactivated and non-inactivated FBS samples were conducted. The results confirmed the absence of mycoplasma contamination in all samples (Data not shown).

### 3.2 FBS quantification of vitamins, hormones, lipids, proteins, ions, carbohydrates, and organic compounds

The complete analysis of all 58 biochemical components in FBS is provided in the supplementary material (SM.Table 1 and SM.Table 2). Below, we present the results for the components that exhibited a coefficient of variation (CV) greater than 15 % in both inactivated and non-inactivated samples (Table 2).

**Table 2:** Quantitative description of biochemical components that showed a coefficient of variation greater than 15 % for non-inactivated FBS samples. Values are expressed as a mean of analysis conducted in triplicate for each sample.

Parameters	Units	FBS 1 (Brazil) Supplier A	FBS 2 (Brazil) Supplier B	FBS 3 (USA) Supplier A	Coefficient of Variation	P value summary
Folic acid	ng/mL	9.80	12.37	4.90	34 %	<0.0001 ^⁎⁎⁎⁎^
Adrenocorticotropic hormone	pg/mL	1.00	4.10	2.93	48 %	0.0593
Anti-thyroglobulin antibody	UI/mL	0.09	0.07	0.06	19 %	0.7732
Direct bilirubin	mg/dL	0.10	0.10	0.06	22 %	<0.0001 ^⁎⁎⁎⁎^
Indirect bilirubin	mg/dL	0.15	0.18	0.22	16 %	0.6645
Iron binding capacity - latent	mcg/dL	49.67	84.27	80.67	22 %	0.0527
Estradiol	pg/mL	19.67	13.00	23.00	22 %	0.0016 ^⁎⁎^
Alkaline phosphatase	U/L	293.67	394.00	209.67	25 %	<0.0001 ^⁎⁎⁎⁎^
Gamma-glutamyl transpeptidase	U/L	5.67	7.33	4.67	19 %	0.1441
LDL	mg/dL	10.33	12.67	7.33	22 %	0.6304
Luteinizing hormone	mUI/mL	0.00	0.01	0.00	102 %	0.506
Parathyroid hormone	pg/mL	19.33	51.67	95.00	56 %	<0.0001 ^⁎⁎⁎⁎^
Total T3	ng/mL	0.82	0.71	1.08	18 %	<0.0001 ^⁎⁎⁎⁎^
Glutamic pyruvic transaminase	UI/L	9.67	9.33	5.67	22 %	0.4195
Transferrin	mg/dL	2.67	2.00	0.0	73 %	0.0490 *
Vitamin C	mg/L	0.05	0.06	0.11	37 %	0.0678

Values represent the mean concentration of each biochemical component analyzed in triplicate (*n* = 3) for each FBS sample. Coefficient of variation (CV) was calculated across the three samples. Statistical analysis was performed using one-way ANOVA. *p* < 0.05 (*), *p* < 0.01 (^⁎⁎^), *p* < 0.0001 (^⁎⁎⁎⁎^).

Regarding non-inactivated FBS samples, 20 out of the 58 biochemical parameters analyzed showed significant heterogeneity across different products, with concentration variations ranging from 16 % to 102 % (Table 2 and SM.Table 1). The components with the highest variations, exceeding 50 %, were adrenocorticotropic hormone, anti-thyroglobulin antibody, parathyroid hormone, and transferrin. The most variable parameters were luteinizing hormone, with a CV of 102 %, and transferrin, with a CV of 73 %.

After heat inactivation, 19 out of the 58 biochemical parameters displayed heterogeneity across the different samples, with concentration variations ranging from 16 % to 84 % (Table 3 and SM.Table 2). The parameters with the highest variability were luteinizing hormone (56 %) and transferrin (84 %). Notably, the inactivation process led to a reduction in the concentrations of anti-thyroglobulin antibody and parathyroid hormone.

**Table 3:** Quantitative description of biochemical components that showed a coefficient of variation greater than 15 % for heat-inactivated FBS samples. Values are expressed as a mean of analysis conducted in triplicate for each sample.

Parameters	Units	FBS 1 (Brazil) Supplier A	FBS 2 (Brazil) Supplier B	FBS 3 (USA) Supplier A	Coefficient of Variation	P value summary
Folic acid	ng/mL	8.00	9.77	4.53	29 %	<0.0001 ^⁎⁎⁎⁎^
Amylase	U/L	25.00	15.33	18.67	20 %	0.0056 ^⁎⁎^
Anti-thyroglobulin antibody	UI/mL	0.05	0.13	0.09	37 %	0.4383
Direct bilirubin	mg/dL	0.08	0.10	0.07	16 %	0.0046 ^⁎⁎^
Iron fixing capacity latent	mcg/dL	53.00	77.00	95.00	23 %	<0.0001 ^⁎⁎⁎⁎^
Estradiol	pg/mL	19.33	15.33	29.00	27 %	<0.0001 ^⁎⁎⁎⁎^
Alkaline phosphatase	U/L	419.00	350.67	148.67	37 %	<0.0001 ^⁎⁎⁎⁎^
Gamma-glutamyl transpeptidase	U/L	6.00	7.33	5.00	16 %	0.0898
Insulin	uU/mL	0.27	0.40	0.43	20 %	0.0110 *
LDL	mg/dL	15.33	9.67	11.67	19 %	0.6032
Luteinizing hormone	mUI/mL	0.01	0.02	0.00	56 %	0.3264
Parathyroid hormone	pg/mL	14.67	60.33	70.33	50 %	<0.0001 ^⁎⁎⁎⁎^
Glutamic pyruvic transaminase	UI/L	5.33	7.67	5.00	20 %	0.4328
Glutamic oxaloacetic transaminase	U/L	28.67	30.67	40.67	16 %	0.0010 ^⁎⁎^
Transferrin	mg/dL	4.33	0.00	2.00	84 %	<0.0001 ^⁎⁎⁎⁎^
Triglycerides	mg/dL	92.67	56.00	58.00	24 %	0.3873
Vitamin C	mg/L	0.09	0.09	0.06	18 %	0.6094
Vitamin D3	ng/mL	18.37	20.13	13.27	17 %	<0.0001 ^⁎⁎⁎⁎^

Values represent the mean concentration of each biochemical component analyzed in triplicate (*n* = 3) for each FBS sample. Coefficient of variation (CV) was calculated across the three samples. Statistical analysis was performed using one-way ANOVA. *p* < 0.05 (*), *p* < 0.01 (^⁎⁎^), *p* < 0.0001 (^⁎⁎⁎⁎^).

Additionally, we compared the mean concentrations of biochemical components between inactivated and non-inactivated samples to assess the impact of heat inactivation on composition. As shown in Table 4, although only insulin exhibited a statistically significant difference between non-inactivated and heat-inactivated samples, the inactivation process tended to alter the concentrations of several components, such as amylase, alkaline phosphatase, and parathyroid hormone.

**Table 4:** Comparison of biochemical components with the highest coefficient of variation in heat-inactivated and non-inactivated FBS samples.

Parameters	Units	Heat-inactivated	Non-inactivated	% Change	P value summary
Alkaline phosphatase	U/L	306.11 ± 114.77	299.11 ± 75.35	−2.34	0.946
Amylase	U/L	19.67 ± 4.01	29.44 ± 4.52	33.19	0.0841
Anti-thyroglobulin antibody	UI/mL	0.09 ± 0.03	0.07 ± 0.01	−28.57	0.5371
Direct bilirubin	mg/dL	0.08 ± 0.01	0.09 ± 0.02	11.11	0.845
Estradiol	pg/mL	21.22 ± 5.74	18.56 ± 4.16	−14.33	0.6232
Folic acid	ng/mL	7.43 ± 2.17	9.02 ± 3.10	17.63	0.5846
Gamma-glutamyl transpeptidase	U/L	6.11 ± 0.96	5.89 ± 1.10	−3.74	0.841
Glutamic oxaloacetic transaminase	U/L	33.33 ± 5.25	35.11 ± 4.79	5.07	0.7421
Glutamic pyruvic transaminase	UI/L	6.00 ± 1.19	8.22 ± 1.81	27.01	0.2202
Insulin	uU/mL	0.37 ± 0.07	0.60 ± 0.07	38.33	0.0304 *
Iron fixing capacity latent	mcg/dL	75.00 ± 17.20	71.53 ± 15.53	−4.85	0.843
LDL	mg/dL	12.22 ± 2.35	10.11 ± 2.18	−20.87	0.4038
Parathyroid hormone	pg/mL	48.44 ± 24.23	55.33 ± 31.00	12.45	0.8166
Transferrin	mg/dL	2.11 ± 1.77	1.56 ± 1.13	−35.26	0.7285
Triglycerides	mg/dL	68.89 ± 16.83	60.33 ± 2.42	−14.19	0.5161
Vitamin C	mg/L	0.08 ± 0.01	0.07 ± 0.03	−14.29	0.7676
Vitamin D3	ng/mL	17.26 ± 2.91	17.57 ± 1.20	1.76	0.896

Data represent mean ± standard deviation of pooled values from the three FBS samples (Brazil, USA, and Paraguay each analyzed in technical triplicate. The aim of this analysis was to compare the overall effect of heat inactivation across samples. Statistical analysis was performed using *t*-test. *p* < 0.05 (*).

### 3.3 FBS quantification of growth factors: EGF, IGF-1, bFGF, and VEGF-A

Quantification of the growth factors EGF, IGF-1, bFGF, and VEGF was performed on lyophilized FBS samples. The analyses showed that EGF and IGF-1 concentrations were below the quantification limits of the respective assay kits, with thresholds of 1.2 ng/mL and 0.82 pg/mL, respectively, as defined by the standard curves provided with each kit.

In contrast, the concentrations of bFGF and VEGF-A fell within the expected ranges according to the standard curves for both untreated and inactivated samples (Table 5). However, the heat inactivation process resulted in a significant concentration reduction of these growth factors, with decreases exceeding 50 % in most cases. Additionally, variability was observed among different products, both for untreated and inactivated samples, indicating intra- and inter- product variability.

**Table 5:** Quantification of bFGF and VEGF-A in inactivated and non-inactivated of the FBS samples.

Sample	bFGF Not Inactivated (pg/mL)	bFGF Inactivated (pg/mL)	bFGF % Change	VEGF-A Not Inactivated (ng/mL)	VEGF-A Inactivated (ng/mL)	VEGF-A % Change
FBS 1 (Brazil) Supplier A	3.74	1.82	51.34	21.24	7.89	62.85
FBS 2 (Brazil) Supplier B	3.47	0.59	83.0	14.36	20.28	41.23
FBS 3 (Brazil) Supplier C	*	1.4	—	*	20.85	—
FBS 4 (USA) Supplier A	4.2	0.0	100.0	19.22	19.74	2.71
FBS 5 (USA) Supplier B	2.73	2.04	25.27	15.18	6.43	57.64
FBS 6 (Paraguay) Supplier A	4.71	1.53	67.52	20.16	18.78	6.85

⁎ This product was only provided in an inactivated form from the supplier. Values represent growth factor concentrations measured in lyophilized and reconstituted FBS samples (*n* = 3). One sample (FBS 6) was only available in inactivated form, as provided by the supplier. Negative values indicate a reduction after inactivation. “—” indicates data not available.

## 4 Discussion

In the early days of the development of *in vitro* cell methodologies the use of FBS was incorporated, as a universal supplement for different cell types, tissues, and organs, from human and animal origin (Gstraunthaler, 2003; Puck, 1959). The cellular effects of FBS constituents include cell proliferation, transport of nutrients, cell adhesion, pH, osmolarity, among others (Van der Valk et al. 2004). However, issues regarding unknown composition and unusual cellular behavior are constant aspects of concern. In this work we studied the biochemical composition of different FBS products, from different local, and different suppliers. The selection of the three FBS products was based on their commercial availability and cost-effectiveness for the study. These factors were crucial for ensuring feasibility and reproducibility within the scope of the experimental design. We also investigated the main nutrients, hormones, and growth factors concentration in both non-inactivated and heat-inactivated samples and the presence of mycoplasma.

Although, our study focused on the detection of mycoplasma, which came out negative in all evaluated samples, it is important to note that FBS can also carry other biological risks, including viruses such as bovine viral diarrhea virus (BVDV), parvoviruses, retroviruses, as well as prions (Wessman, Levings, 1999). To prevent this, despite regulatory bodies and manufacturers implement filtration and inactivation steps, there is no absolute guarantee of sterility (Van der Valk et al. 2004). Such considerations further highlight the urgency of transitioning toward serum-free, chemically defined culture systems to ensure biosafety and reproducibility *in vitro* science.

FBS is a complex mixture of molecules, of different molecular weights, and cellular effects (Brunner, 2010). The main reason for the use of FBS is the unspecific functionality for stimulating cell growth and physiological behavior (Brunner, 2010). The use of this supplement has been considered superior to the other sera due to the low concentration of antibodies and the high growth factors concentrations (Rauch et al. 2011).

Given the biological origin of FBS, its composition is influenced by several factors, including the animal's nutritional status, breed, health condition, age, and the manufacturing process (Wessman and Levings, 1999). In this study, we demonstrated compositional differences among the analyzed products, both in heat-inactivated and non-inactivated forms. Honn et al. (1975) similarly reported substantial variability across FBS samples in parameters such as osmolarity, creatine phosphokinase, lactate dehydrogenase, growth hormone, and prolactin concentrations.

To mitigate the undesirable effects of immune components in serum, heat inactivation is commonly employed. This process can impact in the biochemical constituents and growth factors concentration (Coecke et al. 2005). Our results demonstrate that the inactivation process (heating at 56 °C for 30 min) impacted in the concentration of some constituents, especially regarding the insulin hormone. According to Giard (1987), the FBS heating process decreases the growth rate and cell adhesion (Giard, 1987). Contrasting, Rizzo et al. (1984) suggested that the heating process of the FBS decreases proteolytic activity, which would result in interference in cell proliferation (Rizzo et al. 1984).

Our results demonstrate that the heating process alters the composition of FBS, particularly for heat-sensitive components. For example, insulin concentration showed a significant reduction (Table 4), and both bFGF and VEGF-A levels decreased by over 50 % in most samples (Table 5). These growth factors play central roles in cell proliferation, and survival, suggesting that inactivated FBS may offer relatively lower support for cell growth. Although our study did not include functional assays such as cell proliferation or viability measurements, these aspects can be considered for further studies.

It is important to note that our study was not designed to establish reference concentrations for the growth factors analyzed, nor to benchmark these values against published literature. Instead, our goal was to investigate the variability in growth factor content among different commercially available FBS products. We acknowledge that absolute values reported here may differ from those found in other studies due to variations in detection methods, sample handling, and lyophilization protocols. Nonetheless, the relative comparisons performed under standardized conditions highlight significant inter-product differences that may impact the reproducibility of *in vitro* assays.

Previous proteomic analyses, such as those by Zheng et al. (2006), have demonstrated significant variability in protein composition between FBS lots, including growth factors and transport proteins. Our data also corroborates the concerns raised by Coecke et al. (2005) regarding the impact of serum variability on cell culture reproducibility. The wide range observed for enzymes like alkaline phosphatase and hormones such as LH indicates that even within certified batches, biochemical consistency is not guaranteed.

Among the analytes measured, LH and TF exhibited the highest degree of variability between FBS samples. LH plays a crucial role in reproductive endocrinology and neurological diseases (Webber et al. 2007). Similarly, TF is central to iron metabolism and is involved in cellular processes relevant to anemia, cancer progression, and neurological disorders (Torti and Torti, 2013). The observed differences could influence cellular responses in such models.

Absolute reference values for components in FBS are surprisingly scarce and inconsistent across the literature. Most published studies focus on proteomic composition or relative abundance rather than specific quantification. Furthermore, manufacturer datasheets provide highly variable reference ranges, often without full methodological disclosure. This reinforces our main point: batch-to-batch variability, even within the expected ranges, can significantly affect reproducibility of experimental outcomes.

Besides the ethical concerns, the absence of clear criteria of composition as a parameter of quality of FBS products probably might end up leading to high costs in the *in vitro* research, especially in the biopharmaceutical industry. As described by Baker (2016) less than a third of the methodologies and results described in biomedical articles are reproducible. Such inconsistencies in serum-derived supplements have been widely recognized as a source of poor interlaboratory duplicability in pharmacological and toxicological testing (Hirsch and Schildknecht, 2019).

Recent studies have described promising chemically defined and animal-free culture media, where the exact concentration of components is disclosed, allowing full traceability and standardization of experimental conditions. For example, a protocol published by Weber et al. (2024) describes the formulation of a human-relevant, FBS-free culture medium tailored for normal and tumor cells, with completely defined composition and reproducible results. The availability of such alternatives represents a significant step forward in addressing the limitations of FBS and supports the feasibility of transitioning toward serum-free *in vitro* systems.

Further studies could better explore different batches per supplier. Our approach enabled a controlled assessment of inter-vendor and inter-country variability but did not capture batch-to-batch variation within suppliers. Including multiple lots would strengthen both intra- and inter-vendor comparisons; however, given the exploratory scope, we prioritized broader vendor and geographic coverage to highlight systematic compositional differences among FBS products.

Our findings provide detailed evidence of the biochemical heterogeneity across different FBS products and highlight how even standard procedures like heat inactivation can significantly alter the concentration of key components. As the scientific community moves toward NAMs, the standardization and transparency of culture conditions become critical. Our results reinforce the need for serum alternatives that align with both scientific robustness and ethical imperatives.

## Declaration of generative AI and AI-assisted technologies in the writing process

During the preparation of this work the authors used the ChatGPT Open AI in order to improve the language and readability of the manuscript. After using this tool, the authors reviewed and edited the content as needed and take full responsibility for the content of the publication.

## CRediT authorship contribution statement

**Ana Clara Silva Stival:** Writing – original draft, Methodology, Investigation, Formal analysis, Data curation, Conceptualization. **Artur Christian Garcia da Silva:** Writing – review & editing, Writing – original draft, Supervision, Data curation, Conceptualization. **Marize Campos Valadares:** Writing – review & editing, Writing – original draft, Visualization, Validation, Supervision, Resources, Project administration, Funding acquisition, Formal analysis, Conceptualization.

## Declaration of competing interest

The authors declare that they have no known competing financial interests or personal relationships that could have appeared to influence the work reported in this paper.

## Data Availability

Data will be made available on request.

## Appendix Supplementary materials

Image, application 1
