# Supplementary material for: Qualitative and quantitative evaluation of Fetal Bovine Serum composition: toward ethical and best quality in vitro science
Source: NAM J. 2025 Sep 7;1:100047. doi: 10.1016/j.namjnl.2025.100047 (PMC13289150; doi:10.1016/j.namjnl.2025.100047)
Supplement: Supplementary file 1 [file mmc1.docx]

1. **Supplementary Material**

**SM.Table 1:** Quantitative description of all the 58 biochemical components evaluated in this work for the non-inactivated FBS samples. Values are expressed as a mean ± SD for each sample analyzed in triplicate.

| **Parameters** | **Units** | **FBS 1**  **(Brazil)**  **Supplier A** | **FBS 2**  **(Brazil)**  **Supplier B** | **FBS 3**  **(USA)**  **Supplier A** | **Mean** | **Coefficient of Variation** |
| --- | --- | --- | --- | --- | --- | --- |
| 17- alpha hydroxy- progesterone | ng/dL | 10.67 ± 1.15 | 10 ± 0.00 | 13.67 ± 2.08 | 11.44 | 14% |
| Adrenocorticotropic hormone | pg/mL | 1 ± 0.00 | 4.1 ± 0.46 | 2.93 ± 2.12 | 2.68 | 48% |
| Albumin | g/dL | 1.8 ± 0.00 | 1.9 ± 0.00 | 1.77 ± 0.06 | 1.82 | 3% |
| Alkaline phosphatase | U/L | 293.67 ± 20.21 | 394 ± 19.05 | 209.67 ± 5.03 | 299.11 | 25% |
| Ammonia | umol/L | 642.33 ± 39.26 | 566 ± 2.65 | 673.33 ± 6.43 | 627.22 | 7% |
| Amylase | U/L | 28.67 ± 0.58 | 24.33 ± 3.06 | 35.33 ± 2.89 | 29.44 | 15% |
| Anti-thyroglobulin antibody | UI/mL | 0.09 ± 0.03 | 0.07 ± 0.08 | 0.06 ± 0.06 | 0.07 | 19% |
| Basal Cortisol | micro/dL | 0.00 | 0.00 | 0.00 | 0.00 | * |
| Calcium | mg/dL | 14.9 ± 0.20 | 13.1 ± 0.20 | 12.1 ± 0.20 | 13.37 | 9% |
| Chlorine | meq/L | 102.67 ± 1.15 | 106.33 ± 5.77 | 111.67 ± 12.34 | 106.89 | 3% |
| Cholinesterase | mg/dL | 1500 ± 0.00 | 1500 ± 0.00 | 1500 ± 0.00 | 1500.00 | 0% |
| Direct bilirubin | mg/dL | 0.10 ± 0.01 | 0.1 ± 0.00 | 0.06 ± 0.00 | 0.08 | 22% |
| Estradiol | pg/mL | 19.67 ± 2.52 | 13 ± 1.73 | 23± 1.00 | 18.56 | 22% |
| Fasting blood glucose | mg/dL | 36.33 ± 0.58 | 28 ± 1.00 | 28.83 ± 37.23 | 31.06 | 12% |
| Folic acid | ng/mL | 9.8 ± 0.17 | 12.37 ± 0.15 | 4.9 ± 0.30 | 09.02 | 34% |
| Follicle stimulating hormone | mIU/mL | 0.05 ± 0.00 | 0.05 ± 0.00 | 0.05 ± 0.00 | 0.05 | 0% |
| Fructosamine | mUI/mL | 208 ± 4.58 | 212.33 ± 0.00 | 226.33 ± 0.58 | 215.56 | 4% |
| Globulin | g/dL | 2 ± 0.00 | 1.93 ± 0.06 | 1.9 ± 0.00 | 1.94 | 2% |
| Glutamic oxalacetic transaminase | U/L | 30.33 ± 1.15 | 33.33 ± 3.06 | 41.67 ± 1.53 | 35.11 | 14% |
| Growth hormone | ng/mL | 0.05 ± 0.00 | 0.05 ± 0.00 | 0.05 ± 0.00 | 0.05 | 0% |
| GT gama | U/L | 5.67 ± 0.56 | 7.33 ± 2.31 | 4.67 ± 0.58 | 5.89 | 19% |
| HDL | mg/dL | 10.33 ± 0.58 | 12 ± 1.0 | 11 ± 0.00 | 11.11 | 6% |
| Indirect bilirubin | mg/dL | 0.15 ± 0.13 | 0.18 ± 0.01 | 0.22 ± 0.09 | 0.18 | 16% |
| Insulin | uU/mL | 0.63 ± 0.12 | 0.5 ± 0.10 | 0.6 ± 0.29 | 0.60 | 12% |
| Iron | Ug/dL | 208.67 ± 20.82 | 219 ± 14.42 | 177 ± 16.82 | 201.56 | 9% |
| Iron Fixing Capacity - saturation | mcg/dL | 80.93 ± 6.82 | 70.4 ± 0.72 | 68.47 ± 4.26 | 73.27 | 7% |
| Iron fixing capacity - total | mcg/dL | 258.33 ± 20.30 | 277.67 ± 62.82 | 257.67 ± 9.87 | 264.56 | 4% |
| Iron holding capacity - latent | mcg/dL | 49.67 ± 20.43 | 84.27 ± 13.01 | 80.67 ± 8.02 | 71.53 | 22% |
| Lactic acid | mmol/L | 18.93 ± 0.00 | 17.87 ± 0.46 | 18.23 ± 0.06 | 18.34 | 2% |
| Lactic dehydrogenase | U/L | 1404.67 ± 14.84 | 1608.33 ± 3.79 | 1382 ± 9.85 | 1465.00 | 7% |
| LDL | mg/dL | 10.33 ± 6.03 | 12.67 ± 2.52 | 7.33 ± 9.29 | 10.11 | 22% |
| Lipase | UI/L | 5.33 ± 0.58 | 5.33 ± 0.58 | 5 ± 0.00 | 5.22 | 3% |
| Luteinizing hormone | UI/L | 0 ± 0.00 | 0.01 ± 0.02 | 0.00 ± 0.01 | 0.01 | 102% |
| Magnesium | mg/dL | 3.26 ± 0.25 | 3.09 ± 0.23 | 2.95 ± 0.05 | 3.10 | 4% |
| Match | mg/dL | 10.8 ± 0.40 | 10.87 ± 0.35 | 11.93 ± 0.42 | 11.20 | 5% |
| Osmolarity | mOsm/kg | 261.33 ± 1.15 | 260.33 ± 1.15 | 254.67 ± 1.15 | 258.78 | 1% |
| Parathormone | pg/mL | 19.33 ± 1.15 | 51.67 ± 10.69 | 95 ± 1.00 | 55.33 | 56% |
| Potassium | ng/mL | 10.23 ± 0.40 | 9.63 ± 0.06 | 9.6 ± 0.00 | 9.82 | 3% |
| Progesterone | ng/mL | 0.1 ± 0.00 | 0.1 ± 0.00 | 0.1 ± 0.00 | 0.10 | 0% |
| Prolactin | ng/mL | 0.42 ± 0.31 | 0.6 ± 0.00 | 0.6 ± 0.00 | 0.54 | 16% |
| Pyruvial glutamate transaminase | UI/L | 9.67 ± 5.51 | 9.33 ± 1.15 | 5.67 ± 3.51 | 8.22 | 22% |
| Serum creatinine | mg/dL | 3.19 ± 0.09 | 3.03 ± 0.02 | 2.48 ± 0.07 | 2.90 | 10% |
| Sodium | mmol/L | 131.67 ± 0.58 | 136.67 ± 0.58 | 130 ± 0.00 | 132.78 | 2% |
| T3 total | ng/mL | 0.82 ± 0.02 | 0.71 ± 0.02 | 1.08 ± 0.05 | 0.87 | 18% |
| T4 - Thyroxine | mcg/dL | 11.69 ± 0.29 | 13.01 ± 0.53 | 12.85 ± 0.54 | 12.52 | 5% |
| T4 Free | mcg/dL | 1.78 ± 0.04 | 1.89 ± 0.03 | 1.84 ± 0.04 | 1.84 | 3% |
| Total bilirubin | mg/dL | 0.25 ± 0.13 | 0.26 ± 0.01 | 0.28 ± 0.09 | 0.26 | 4% |
| Total cholesterol and fractions | mg/dL | 30 ± 9.54 | 38.67 ± 3.51 | 32.33 ± 10.12 | 33.67 | 11% |
| Total proteins | g/dL | 3.83 ± 0.06 | 3.8 ± 0.00 | 3.67 ± 0.06 | 3.77 | 2% |
| Transferrin | mg/dL | 2.67 ± 1.53 | 2 ± 1.00 | 0 ± 0.00 | 1.56 | 73% |
| Triglycerides | mg/dL | 59.33 ± 2.08 | 58 ± 1.73 | 63.67 ± 5.51 | 60.33 | 4% |
| Urea | mg/dL | 36.33 ± 1.15 | 37.33 ± 2.52 | 33 ± 0.00 | 35.56 | 5% |
| Uric acid | mg/dL | 1.33 ± 0.00 | 1.47 ± 0.00 | 1.67 ± 0.49 | 1.49 | 9% |
| Vitamin A | mg/L | 0.17 ± 0.06 | 0.2 ± 0.00 | 0.2 ± 0.00 | 0.19 | 8% |
| Vitamin B12 | pg/mL | 277 ± 7.94 | 253.67 ± 10.02 | 240 ± 24.25 | 256.89 | 6% |
| Vitamin C | mg/L | 0.05 ± 0.00 | 0.06 ± 0.01 | 0.11 ± 0.05 | 0.07 | 37% |
| Vitamin D3 | ng/mL | 18.33 ± 0.38 | 18.5 ± 0.00 | 15.87 ± 0.25 | 17.57 | 7% |
| Vitamin E | mg/L | 0.5 ± 0.00 | 0.5 ± 0.00 | 0.5± 0.00 | 0.50 | 0% |

**SM.Table 2:** Quantitative description of all the 58 biochemical components evaluated in this work for the heat-inactivated FBS samples. Values are expressed as a mean ± SD for each sample analyzed in triplicate.

| **Parameters** | **Units** | **FBS 1**  **(Brazil)**  **Supplier A** | **FBS 2**  **(Brazil)**  **Supplier B** | **FBS 3**  **(USA)**  **Supplier A** | **Mean** | **Coefficient of variation (CV)** |
| --- | --- | --- | --- | --- | --- | --- |
| Albumin | g/dL | 1.8 ± 0.00 | 1.9 ± 0.00 | 1.8 ± 0.00 | 1.83 | 3 % |
| Globulin | g/dL | 2 ± 0.00 | 1.67 ± 0.06 | 1.87 ± 0.06 | 1.94 | 3 % |
| 17-alpha hydroxy-progesterone | ng/dL | 14.33 ± 7.51 | 16.33 ± 0.85 | 13.33 ± 5.77 | 14.67 | 9 % |
| Folic acid | ng/mL | 8 ± 0.26 | 9.77 ± 0.85 | 4.53 ± 0.23 | 7.43 | 29 % |
| Lactic acid | ng/mL | 19.27 0.55 | 19.13 ± 0.00 | 17.3 ± 2.55 | 18.57 | 5 % |
| ACTH - adrenocorticotropic hormone | pg/mL | 1 ± 0.00 | 1 ± 0.00 | 1 ± 0.00 | 1.00 | 0 % |
| Amylase | U/L | 25 ± 1.00 | 15.33 ± 0.58 | 18.67 ± 3.79 | 19.67 | 20 % |
| Ammonia | umol/L | 668 ± 12.17 | 573.67 ± 2.08 | 680 ± 3.61 | 640.56 | 7 % |
| Anti-thyroglobulin antibody | UI/mL | 0.05 ± 0.09 | 0.13 ± 0.09 | 0.09 ± 0.03 | 0.09 | 37 % |
| Direct bilirubin | mg/dL | 0.083 ± 0.01 | 0.1 ± 0.00 | 0.07± 0.03 | 0.08 | 16 % |
| Indirect bilirubin | mg/dL | 0.17 ± 0.04 | 0.17 ± 0.07 | 0.16 ± 0.01 | 0.17 | 2 % |
| Total bilirubin | mg/dL | 0.25 ± 0.04 | 0.24 ± 0.05 | 0.23 ± 0.01 | 0.24 | 4 % |
| Calcium | mg/dL | 13.3 ± 0.10 | 14.27 ± 0.32 | 12.63 ± 0.12 | 13.40 | 5 % |
| Iron binding capacity - latent | mcg/dL | 53 ± 5.29 | 77 ± 2.00 | 95 ± 5.57 | 75.00 | 23 % |
| Iron binding capacity - saturation | mcg/dL | 79.8 ± 1.73 | 71.53 ± 0.45 | 64.07 ± 2.51 | 71.80 | 9 % |
| Iron binding capacity - total | mcg/dL | 259.33 ± 18.50 | 269.67 ± 6.35 | 264.33 ± 4.51 | 264.44 | 2 % |
| Chlorine | meq/L | 101.33 ± 1.15 | 112.33 ± 6.35 | 105.67 ± 1.53 | 106.44 | 4 % |
| Total cholesterol and fractions | mg/dL | 25.67 ± 4.73 | 30.67 ± 7.64 | 35.67 ± 9.29 | 30.67 | 13 % |
| Cholinesterase | mg/dL | 1500 ± 0.00 | 1500 ± 0.00 | 1500 ± 0.00 | 1500.00 | 0 % |
| Basal cortisol | micro/dL | 0.00 | 0.00 | 0.00 | 0.00 | * |
| Serum creatinine | mg/dL | 3.09 ± 0.09 | 3.38 ± 0.13 | 2.42 ± 0.05 | 2.96 | 14 % |
| Estradiol | pg/mL | 19.33 ± 0.58 | 15.33 ± 1.53 | 29 ± 2.0 | 21.22 | 27 % |
| Iron | Ug/dL | 206.33 ± 14.84 | 193.33 ± 4.51 | 169.33 ± 8.96 | 189.67 | 8 % |
| Alkaline phosphatase | U/L | 419 ± 2.65 | 350.67 ± 4.04 | 148.67 ± 7.37 | 306.11 | 37 % |
| Phosphorus | mg/mL | 10.2 ± 0.26 | 10.57 ± 0.21 | 11.1± 0.44 | 10.62 | 3 % |
| Fructosamine | mUI/mL | 208.33 ± 2.08 | 211.33 | 225.33 ± 8.50 | 215.00 | 3 % |
| Follicle stimulating hormone | mIU/mL | 0.05 ± 0.00 | 0.05 ± 3.51 | 0.05 ± 0.00 | 0.05 | 0 % |
| Gamma gt | U/L | 6 ± 1.00 | 7.33 ± 1.15 | 5 ± 1.0 | 6.11 | 16 % |
| Growth hormone | ng/mL | 0.05 ± 0.00 | 0.05 ± 0.00 | 0.05 ± 0.00 | 0.05 | 0 % |
| Fasting blood glucose |  | 35.33 ± 1.53 | 28 ± 0.00 | 69 ± 1.00 | 44.11 | 40 % |
| HDL | mg/dL | 10.33 ± 0.58 | 11.67 ± 0.58 | 10 ± 0.00 | 10.67 | 7 % |
| Insulin | mU/mL | 0.27 ± 0.06 | 0.4 ± 0.00 | 0.43 ± 0.06 | 0.37 | 20 % |
| Dehydrogenase lactic | U/L | 1354 ± 20.66 | 1471.33 ± 8.50 | 1315 ± 14.00 | 1380.11 | 5 % |
| LDL | mg/dL | 15.33 ± 4.93 | 9.67 ± 4.93 | 11.67 ± 9.29 | 12.22 | 19 % |
| Luteinizing hormone | UI/L | 0.01 ± 0.01 | 0.02 ± 0.02 | 0.00 ± 0.01 | 0.01 | 56 % |
| Lipase | UI/L | 4.67± 0.58 | 4 ± 0.00 | 4 ± 0.00 | 4.22 | 7 % |
| Magnesium | mg/dL | 3.16 ± 0.03 | 2.93 ± 0.08 | 2.80 ± 0.03 | 2.96 | 5 % |
| Osmolarity | mOsm/kg | 260 ± 2.00 | 265 ± 0.00 | 259 ± 2.00 | 261.33 | 1 % |
| Parathyroid hormone | pg/mL | 14.67 ± 0.58 | 60.33 ± 2.08 | 70.33 ± 4.04 | 48.44 | 50 % |
| Potassium | ng/mL | 9.83 ± 0.06 | 9.4 ± 0.00 | 9.7 ± 0.20 | 9.64 | 2 % |
| Progesterone | ng/mL | 0.1 ± 0.00 | 0.1 ± 0.00 | 0.1 ± 0.00 | 0.10 | 0 % |
| Prolactin | ng/mL | 0.6 ± 0.00 | 0.6 ± 0.00 | 0.6 ± 0.00 | 0.60 | 0 % |
| Total proteins | g/dL | 3.8 ± 0.00 | 3.8 ± 0.00 | 3.67 ± 0.06 | 3.76 | 2 % |
| Uric acid | mg/dL | 1.3 ± 0.00 | 1.2 ± 0.00 | 1.9 ± 2.55 | 1.47 | 21 % |
| Sodium | mmol/L | 135 ± 0.00 | 136 ± 0.00 | 129.67 ± 1.53 | 133.56 | 2 % |
| Total T3 | ng/mL | 0.85 ± 0.03 | 0.71 ± 0.01 | 1.01 ± 0.04 | 0.86 | 14 % |
| T4 - Thyroxine | mcg/dL | 11.57 ± 0.68 | 12.92 ± 0.73 | 11.80 ± 0.10 | 12.10 | 5 % |
| Free T4 | mcg/dL | 1.79 ± 0.01 | 2.01 ± 0.04 | 1.92 ± 0.12 | 1.90 | 5 % |
| Glutamic pyruvic transaminase - TGP | UI/L | 5.33 ± 0.58 | 7.67 ± 0.58 | 5 ± 4.36 | 6.00 | 20 % |
| Glutamic oxaloacetic transaminase - TGO - AST | UI/L | 28.67 ± 1.53 | 30.67 ± 1.53 | 40.67 ± 3.06 | 33.33 | 16 % |
| Transferrin | mg/dL | 4.33 ± 0.58 | 0 ± 0.00 | 2 ± 0.00 | 2.11 | 84 % |
| Triglycerides | mg/dL | 92.67 ± 58.31 | 56 ± 4.00 | 58 ± 3.46 | 68.89 | 24 % |
| Urea | mg/dL | 38 ± 0.00 | 38.33 ± 2.08 | 33.67 ± 3.06 | 36.67 | 6 % |
| Vitamin A | mg/L | 0.25 ± 0.07 | 0.2 ± 0.00 | 0.2 ± 0.00 | 0.22 | 11 % |
| Vitamin B12 | pg/mL | 260.67 ± 8.02 | 259.67 ± 23.01 | 245.33 ± 16.17 | 255.22 | 3 % |
| Vitamin C | mg/L | 0.09 ± 0.06 | 0.09 ± 0.04 | 0.06 ± 0.02 | 0.08 | 18 % |
| Vitamin D3 | ng/mL | 18.37 ± 0.06 | 20.13 ± 0.47 | 13.27 ± 0.38 | 17.26 | 17 % |
| Vitamin E | mg/L | 0.5 ± 0.00 | 0.37 ± 0.12 | 0.43 ± 0.12 | 0.43 | 13 % |
